# Hormesis and Defense of Infectious Disease

**DOI:** 10.3390/ijms18061273

**Published:** 2017-06-15

**Authors:** Sebastian Weis, Ignacio Rubio, Kristin Ludwig, Cynthia Weigel, Elisa Jentho

**Affiliations:** 1 Department of Anesthesiology and Intensive Care Medicine, University Hospital Jena, Jena 07747, Germany; Cynthia.Weigel@med.uni-jena.de (C.W.); elisa.jentho@med.uni-jena.de (E.J.); 2 Center for Infectious Diseases and Infection Control, University Hospital Jena, Jena 07747, Germany; 3 Center for Sepsis Control and Care, University Hospital Jena, Jena 07747, Germany; 4 Institute of Molecular Cell Biology, Center for Molecular Biomedicine (CMB), University Hospital Jena, Jena 07745, Germany; ignacio.rubio@med.uni-jena.de (I.R.); kristin.ludwig@med.uni-jena.de (K.L.); 5 Fritz Lipmann Institute, Leibniz Institute on Aging, Jena 07745, Germany

**Keywords:** tissue damage, tolerance, hormesis, sepsis, DNA damage

## Abstract

Infectious diseases are a global health burden and remain associated with high social and economic impact. Treatment of affected patients largely relies on antimicrobial agents that act by directly targeting microbial replication. Despite the utility of host specific therapies having been assessed in previous clinical trials, such as targeting the immune response via modulating the cytokine release in sepsis, results have largely been frustrating and did not lead to the introduction of new therapeutic tools. In this article, we will discuss current evidence arguing that, by applying the concept of hormesis, already approved pharmacological agents could be used therapeutically to increase survival of patients with infectious disease via improving disease tolerance, a defense mechanism that decreases the extent of infection-associated tissue damage without directly targeting pathogenic microorganisms.

## 1. Introduction

**Figure 1:**
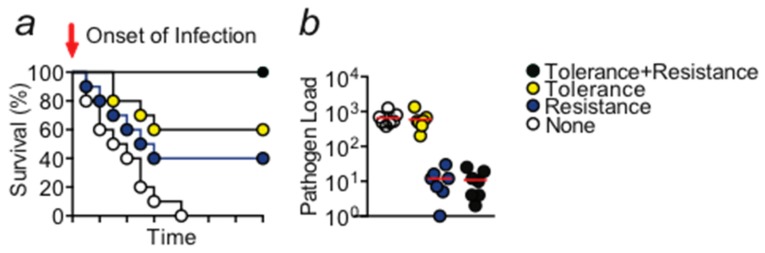
(**a**) Schematic Kaplan–Meier survival plot illustrating the protective effect exerted by disease tolerance and resistance mechanisms. Activity of both mechanisms is required in order to obtain the optimal level of protection. (**b**) Schematic display of the pathogen load of any given organ, animal, or species. A gene that provides disease tolerance to infection acts independently of the pathogen load while a gene that provides resistance acts via reducing the number of pathogens. Circles would indicate individual samples.

Infections are among the leading causes of death, taking the life of 10 to 15 million people every year [1,2]. The majority of fatal cases occur in the developing world [2]. Even in settings with sufficient health care resources, treatment of patients with infections is becoming increasingly difficult due to raising rates of microbial resistance to applied agents affecting mostly protozoan [3] and bacterial [4,5] pathogens. Despite new antimicrobial drugs licensed in recent years, pharmacological innovation is still insufficient. Strategies that have been deployed to decrease infectious disease mortality such as disinfection, vaccination, and antimicrobial drugs share the same mode of action, i.e., the reduction of the number of pathogens [6,7]. This strategy is used by the immune system and in this context referred to as resistance [6,7]. However, infected hosts also use another less recognized defense strategy namely disease tolerance to infection, whose potential for the treatment of infectious disease has been under-recognized. Mechanisms that afford disease tolerance limit the extent of tissue damage associated with infection (tissue damage control (described in [6,8])). In order to obtain an optimized level of protection against infectious disease, activity of both defense mechanisms will be required (Figure 1). Of note, stress or damage to the parenchyma is not only imposed by pathogens or their products, i.e., toxins. Also, soluble and cellular components of the immune system, such as cytokines produced by myeloid cells or lymphocytes, are potentially harmful [9,10]. The dysfunction and damage imposed by immune systems is commonly referred to as immunopathology [7,11], a well described driver of bacterial infections [9,10,12] and sterile inflammation [13,14].

## 2. Disease Tolerance as a Concept of Protection against Inflammatory Tissue Damage

Disease tolerance as an important defense mechanism has been recognized in the field of botany for over 120 years. First descriptions can be traced to an Australian study published in 1894 in which Nathan Augustus Cobb investigated rust-enduring in wheat [15,16] Sixty years later, tolerance was portrayed, as the ability to withstand an infection without loss in fitness [16,17]. While some authors describe the same defense strategy as ‘endurance’ or ‘resilience’ [18], the term tolerance has recently been used explicitly in accordance with immune tolerance, based on a phenomenon that limits damage elicited by a given effector [19]. Conceptually, disease tolerance to infection has been translated into mammals by the pioneering work from groups lead by Read [20], Medzhitov [21], and Soares [22,23,24] and comprehensively reviewed recently [6,7,8]. Overall, disease tolerance has been shown to provide protection against different classes of pathogens, including of viral [25], protozoan [20,23], or bacterial origin [24,26].

Beneficial effects of disease tolerance to infection have been in depth revealed in the field of iron/heme infection biology. Degradation of heme and appropriate adaptive metabolism of released iron are important features that decrease tissue dysfunction/damage and enhance survival in malaria [27,28,29] and polymicrobial sepsis [24,26]. More generally, a variety of master-regulators are held responsible to assure tissue damage control during systemic infections (described in detail in reference [8]). These encompass transcription factor nuclear factor-erythroid 2-related factor 2 (NRF2) for the oxidative stress response [30] or ataxia teleangiectasia mutated (ATM) kinase (ATM) for the DNA damage response [9] amongst others [31].

## 3. Hormesis

“*Dosis sola facit venenum*” 500 years ago, Theophrastus Bombastus von Hohenheim, also known as Paracelsus, described a long-known phenomenon of dose dependent effects of pharmacological active substances also referred to as hormesis (reviewed in [32,33]). Underlying biphasic dose-response relationships have been investigated extensively for immune cell migration, cytokine release, activity, and trafficking (reviewed in [34]). While many of the described drugs are used in the daily routine, their hormetic potential to alter immune cell function and gene transcription in infectious disease or their capability to activate disease tolerance mechanisms are yet unexplored.

The concept of hormesis in pharmacological application for infectious disease has recently been revived due to fundamental discoveries in pharmacology and toxicology applying substances at very low doses. For example, gasotransmitters such as carbon monoxide are commonly known for their strong toxic and lethal effects, causing hypoxia via the formation of complexes with heme (carboxyhemoglobin) [35]. The same gasotransmitters, if administered at lower dosages, protect against the deleterious outcome of endotoxemia [36] and malaria [29].

According, to the concept of hormesis, low-dose preconditioning by pro-inflammatory cues, or other harmful cues, can modify a subsequent response to the same or alternative insults [37,38].

This notion has been investigated in detail recently in the case of adaptation of monocytes to stress. Exposing monocytes to different pathogen associated molecular patterns (PAMPs) at particularly low dosage resulted in an adaptive cytokine release after a subsequent application of bacterial lipopolysaccharide (LPS) [39]. Low concentrations of LPS (10 pg/mL) resulted in increased TNFα and Interleukin-6 levels, whereas high concentrations (10–100 ng/mL) induced immune tolerance as shown by reduced release of TNFα and Interleukin-6 after stimulation [39].

**Figure 2:**
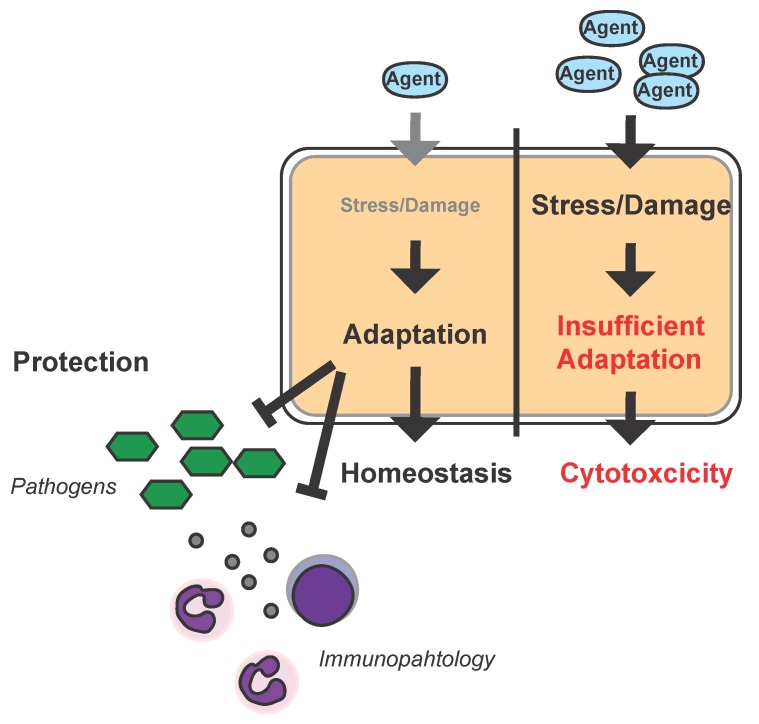
Hormesis and infection: Schematic model of the hormetic response to infection in the parenchyma. High dosages of a given agent (e.g., epirubicin) can impose stress and damage to a cell or organ to an extent that results in insufficient adaptive responses leading to cytotoxicity. Low dosages of the same agent will also impose stress/damage to the cell/organ but allow for adaptation which increases the capacity of the cell/organ to withstand stress/damage imposed by pathogens, their products, i.e., toxins, the immune system (immunopathology), or damage associated molecular patterns such as heme during infections and can in turn provide a pathogen-independent host-targeted approach to treat infectious disease.

Another study that is described in more detail in the next section reported beneficial effects of low-dose chemotherapeutics, i.e., anthracyclines, for the treatment of murine bacterial sepsis. Sepsis is a severe condition arising from a deregulated host response to infection associated with organ failure [40]. Pharmacological activation of damage repair mechanisms represents an interesting approach for new treatment options of infectious diseases, such as sepsis, that could operate by imposing disease tolerance to infection. Activation of stress and damage responses can be achieved by a variety of different agents and conditions. Applying the concept of hormesis, low doses of otherwise harmful agents would activate cytoprotective pathways that should be protective against a secondary, e.g., infectious event exerting cross-tolerance. In concordance with this notion, low doses of toxins or radioactivity can decrease the mortality of septic mice (Figure 2) [9,41]. How far disease tolerance and/or resistance mechanisms are capable of inducing reciprocal adaptation via hormetic mechanisms remains to be established.

## 4. Induction of Disease Tolerance by DNA Damage

Anthracyclines—a class of chemotherapeutic agents in clinical use for over 30 years [42]—have recently been shown to afford tissue damage control in animal models of sepsis [9]. Using a screen of about 2300 pharmacological compounds, three substances belonging to the group of anthracyclines—i.e., epirubicin, doxorubicin, and daunorubicin—were found to decrease the inflammatory response of THP-1 monocytes upon exposure to *Escherichia coli*. Subsequently, when subjected to polymicrobial infection, mortality of mice that were treated with a low dose of any of these three compounds significantly improved survival as compared to control mice that received vehicle only. These effects were not restricted to one mouse strain, as NMRI mice were equally protected, nor to one disease model, as mice subjected to pneumonia by *Klebsiella pneumoniae*, a clinically relevant gram-negative bacterium, were also protected. Epirubicin also protected mice against endotoxin shock induced by high doses of LPS and decreased serum concentrations of inflammatory cytokines and serology markers of organ injury. Based on the observation that the survival benefit was not associated with a decrease in pathogen load in lung, kidney, liver, or spleen of the mice 24 h after infection, the authors concluded that low-dose epirubicin exerted protection against the deleterious outcome of bacterial infection via promoting disease tolerance. Mechanistically, it was shown that protection is attributed to a systemic induction of the DNA damage response (DDR) via activation of the ATM kinase [9]. This is in concordance with previous data, providing evidence that anthracyclines activate the DNA damage response mediator through ATM [43]. In mice with a constitutive deletion of the gene encoding for ATM (*Atm^−/−^*), epirubicin failed to protect against polymicrobial sepsis, providing further evidence for an important role for induction of DDR as an adaptive response.

If activation of the DDR is a general mechanism that prevents sepsis pathology, then activation of the DDR via other means should also exert the same effects as epirubicin. In fact, whole-body γ-irradiation increased survival of mice subjected to polymicrobial sepsis associated with increased ATM activation while tissue specific deletion in the lungs using an adenovirus-mediated approach reverted the protection.

This landmark study is the first to show a beneficial hormetic effect of anthracyclines for the treatment of bacterial infections. The authors suggest low-dose epirubicin as a possible new treatment of septic patients as epirubicin treatment showed an increased survival even if administered 24 h after sepsis induction, when combined with former antibiotic treatment [9], making it not just an opportunity to prevent sepsis, but also a potential therapeutic option. These findings are supported by recent evidence showing that topoisomerase I inhibitors and other chemotherapeutic agents also interact with the DNA-replication improve the outcome of sepsis [41]. These studies exhibit new perspectives in the application of low, hormetic doses of otherwise toxic agents like anthracyclines, as the applied dosages that were protective in the animal studies correspond to only one-fifth of the dosage used in a single cycle of chemotherapy [44]. Therefore, relevant cytotoxicity, such as cardiotoxicity or bone marrow depression in the case of anthracyclines, should not be expected with this approach.

Of note, the same mode of action—i.e., activation of DNA damage responses in response to DNA damage—have been proposed to prevent tumor development and enhance extension (reviewed in [45]). It remains to be elucidated whether accumulation of damage over a longer period (aging) and acute damage imposed by infections evoke similar adaptive cellular responses and whether exhaustion of this damage response system will specifically lead to chronic or acute disease. While therapeutic targeting of the DNA damage response in acute infectious disease in humans can be investigated in short term, targeting the chronic application of substances to prevent tumor development or aging will be more challenging, as (i) this would require repetitive application of substances in hormetic dosages which might breach the hormetic approach and (ii) long-term follow-up in humans will need to be assured.

In sum, the reported benefits of application of low-dose epirubicin in systemic inflammation can be perceived as a first and most promising example of the therapeutic potential of already approved drugs arising from the concept of hormesis.

## 5. Mechanisms Acting Downstream of DNA Damage Control: Autophagy

As outlined above, adaptive responses are not restricted to the DDR but encompass diverse evolutionarily conserved pathways such as the unfolded protein response, heat shock response, or organelle damage response inducing autophagy [8]. Recent publications reveal a role for autophagy as an important regulator of tissue damage control and prevention of immunopathology [7,8,9,10]. Autophagy refers to the process by which damaged or aged cellular components, e.g., cellular organelles, are degraded after formation of autophagolysosomes involving the participation of different proteins such as Beclin-1, the serine/threonine protein kinase ULK1, microtubule-associated protein 1A/1B-light chain 3 (LC3), and others [46]. A role of autophagy in the protective effect of DDR in sepsis was shown using an autophagy-defective (*Lc3b^−/−^*) mouse strain in which epirubicin treatment did not exert any protection against bacterial sepsis. Moreover, lung specific deletion of *Atg7*, another gene involved in autophagy similarly abolished the protective effects of epirubicin. Accordingly, using a gain-of-function adenoviral approach to overexpress *Atg7* increased survival rates in the same animal model [9]. Autophagy also reduced liver injury in models of sterile inflammation [47]. In addition to its reported effects on disease tolerance, autophagy can also affect components of the resistance machinery, namely the immune system, leading to the modulation of inflammasome activation [48], macrophage polarization [49], or the function of adaptive immune cells during infection [50,51], amongst others. Macrophages with a constitutive deletion of *Srbl* (a scavenger receptor class B type 1) which are characterized by maladaptive autophagy, exhibit increased TNFα and IL-6 production in response to LPS [10]. These observations are consistent with the notion that defects in autophagy might compromise tissue damage control and enhance immunopathology. Pharmacological agents that interfere with autophagy pathways have already been licensed and, as in the case of the mammalian target of rapamycin (mTOR) inhibitor rapamycin, are used as potent immunosuppressive drug in organ transplant patients and might additionally possess antitumor properties [52,53]. While there is no direct evidence that mTOR inhibition increases tissue damage control in systemic infections, the available evidence is in support of this approach: (i) application of rapamycin protected tubular epithelial cells against TNFα application [54]; (ii) rapamycin ameliorated LPS induced hypotension and inflammation [55]; (iii) it improved cognitive impairments in sepsis survivors [56]; and (iv) application of rapamycin rescued mice from staphylococcal infections [57]. Other studies have similarly reported the reduction of sepsis associated tissue damage via induction of autophagy [9,58]. Considering the well-established role of mTOR as a negative regulator of autophagy, these and other reports point to the involvement of autophagy in disease tolerance, underlying its general role in cellular homeostasis. Of note, in apparent contradiction to those findings, the activity of kinases lying upstream of mTOR—namely phosphoinositide 3-kinase (PI3K) or AKT—has been proposed to enable tissue damage control to different cellular stress events [8]. However, selective inhibition of mTOR, and not the entire pathway, might shift signal transduction towards enhanced cytoprotection. Indeed, inhibition of mTOR is known to activate PI3K/AKT signaling via the operation of well-established negative feedback loops sparked by mTOR feeding back at the level of PI3K/AKT activation [59,60,61]. As such, mTOR inhibition could provide disease resistance both via a direct stimulation of autophagy and by boosting PI3K/AKT activity by releasing negative feedback mechanisms. Pharmacological activation of genes controlling autophagy might be another attractive approach for the treatment of infectious diseases. It remains to be established as to how far low doses of rapamycin that would not cause immunosuppression will prove useful in improving the outcome of infectious disease and if so, whether this would act via modulating tissue tolerance mechanisms.

## 6. Outlook

The concept of dose dependent distinct dose responses to approved agents possesses large biomedical implications. These extent beyond infectious disease and include new treatment options for cancer, auto-immune and other chronic disorders [34]. In this short review, we have focused on how disease tolerance mechanisms can be altered applying the concept of hormesis. Pharmacological manipulation of mechanisms that enhance tissue damage control and improve or restore disease tolerance is a largely unexplored but innovative approach for the treatment of acute inflammatory disease [8,20,62]. At least theoretically, this approach should have the advantage as compared to antimicrobial agents in that development of resistance against the applied substances does not occur as no selection pressure is exerted on the pathogenic microorganisms [8,62]. A pproved drugs that target damage control pathways are being used for other purposes, such as depicted in detail for anthracyclines in this article and maybe other chemotherapeutic agents [41]. As discussed in this review, the same drugs, administered at low-dosage, might prove beneficial for infectious disease and could be tested in preclinical studies and subsequent clinical trials. Whether any of these substances can also be applied locally—e.g., to specifically induce the cytoprotective responses in the lung in patients with pneumonia—is unknown. This approach should have the advantage of lower systemic side effects. However, it has to be considered that the time window between onset of infection and therapeutic application in this setting might even be shorter, as the drugs would have to be applied before systemic complications occur.

Overall, we expect that the armory against infectious disease can be expanded significantly considering the concept of hormesis and approaching mechanisms that provide disease tolerance to infection.
